# Editorial Chit-Chat

**Published:** 1877-01

**Authors:** 


### Editorial Chit-Chat.

PAY UP.—End of year. Subscription due. Blank enclosed. Only fifty-five cents.

FINE SINGING.—The singing of the Quartette at Park Church is unsurpassed. It is worth the price of admission to a good concert to hear them.

STREET CARS.—The New York papers are complaining of the discomfort from riding in cold street cars. Here in Elmira we have our street cars warmed with snug little stoves.

A VACANCY.—There is a principalship vacant in one of the public schools of our city. Salary $1,500 per year. C. N. Shipman, Esq., Chairman of the Teachers' Committee, is anxious to receive applications for the same.

WM. R. WARNER, & Co., of Phil., have received the Centennial award for their soluble Sugar Coated Pills. This is the third grand World's Fair prize that attests to their excellence over all competition at home and abroad.

HIDEOUS CHROMO.—A new chromo is offered for sale, representing a trout, entwined by an eel and clawed by a miserable boiled lobster. What sentiment—what a poetic conception that, of placing a trout—the beautiful speckled darling of the mountain angler, with a nasty, slimy eel—bah!

FLORICULTURE.—Next to having a felon on your finger, the most entertaining employment is to cultivate flowers. If our premises do not blossom next year, it will not be the fault of Vick's seeds and bulbs that are sprouting in our greenhouse awaiting the time for their transplantation.

JOHNSON.—Who in fury is Johnson? Is it possible that a man can own a collection of paintings which at auction, in these hard times too, brought over four hundred thousand dollars, and yet remain unknown? Mr. Johnson must have possessed a handsome fortune to have invested so much money in art.

PERSONAL—rather, but then it would be a great advantage to the county if we had a few more Supervisors like John D. Williams, of this city. Mr. Williams has *attended* to the duties of his office, and the sequel has shown that much is to be done when the office is faithfully filled. The tax-payers owe Supervisor Williams their thanks for the masterly manner in which he has cared for their interests.

THE DIFFERENCE.—T. K. Beecher, of Elmira, refused a pass from the president of a railroad, recently, because the president was not sole owner of the road, and perhaps to get a notice for excentricity.—*Utica Herald.*

Mr. Beecher was governed in the transaction by what he conceived to be honorable. In this respect he differs widely from the individual who penned the above item.

MICRO-PHOTOGRAPHS.—The micro-photographs in histology, published by Jos. H. Cotes & Co., of Philadelphia, are precisely what have been long needed by the profession. Few physicians have the leisure or apparatus to enter into histological microscopy, yet such investigations are of immense advantage to every scientific physician. The publication referred to, comes very opportune therefore, and we are glad to know that it is having an extensive and deserved circulation.

TIME.—Blank enclosed with which to renew your subscription. The P. O. Department have decided that we cannot enclose our envelope, so that you will be compelled to furnish your own hereafter. Please enclose your fifty-five cents and send us a new subscriber, too, if you can. Nowhere can you find so cheap and useful a journal as the BISTOURY. Look over our table of contents, and see whether or no we do not give you the full worth of your money, in a single number. Pay promptly—the moment the journal is received. Our next issue is likely to surprise you with some contemplated improvements. Wait and see.

CHARITY PATIENTS.—All physicians agree in that charity patients, or those who receive the service of the medical fraternity gratuitously, are invariably the most troublesome and the most exacting of all patients. They never seem to be satisfied with what you do for them, and are the first ones, and usually the only ones, to make ill-natured remarks about you.

They generally require ten times more of your time than those who pay for your attentions, and seem to have no other designated sphere in the economy of nature than to annoy and pester all physicians with whom they come in contact. Whether all impecuneous people are infernal nuisances, or whether all I. N's are impecunious, we do not clearly understand, but that little or no difference exists between them, seems very probable—especially when sick.

---

THE PRESIDENTIAL MUDDLE.—We do hope Congress, or whoever has authority, will get us out of the present difficulty, in which we find ourselves by reason of the closeness of the presidential election, without leading the nation into any internal strife or trouble. We can all afford to sacrifice our preference as to the man we should like to see President, and be contented and prospered under either. Let us keep cool, therefore, and abide the decision of those who have the power to decide for us, and let us hope that in future we may be permitted to vote for President by a popular vote, and so prevent the possibility of another such a muddle.

---

THE ZOO.—Everybody in Philadelphia, who can afford the requisite admission fee, makes a weekly visit to the Zoological Garden, there to spend an hour or two in witnessing the antics of the monkeys and other animals. Well, we went with the rest, and, having made a tour of the grounds, found our way to the gate, at which we entered, to take our departure for a ride upon the Schuylkill. By the side of the entrance gate is a turn style—looking very much like a large cage—and labeled "Exit," through which large numbers of persons were passing upon their way home. A couple of rustic youths had just passed through the entrance gate, and, seeing the crowd pushing toward the turn style, while they beheld the mysterious label "exit" just above, concluded some curious animal was there being interviewed by the crowd, so with a "come Joe, let's see the exit, too," they patiently awaited their turn, and soon found themselves on the outside the grounds, costing them an extra twenty-five cents to be again admitted. Evidently they came to see the elephant, but instead, saw the "exit."

---

A BALANCING EFFORT.—Of all the sad, sad sights that man is called upon to behold, perhaps the most distressing is to witness the effort of a lady in climbing into the upper berth of a sleeping coach, while you occupy the one immediately below. The situation is fraught with so much apprehensiveness to the individual below—a doubt, mingled with undisguised fear that the fair climber may finally loose her balance entirely, while yanking her body back and forth on the outside rail of her destined perch, much after the fashion of a wounded squirrel striving to retain his grasp upon a tree-top, and fall—blue garters and all—upon your prostrate body, in the lower compartment. Then, the reflection that she might light fairly within your own private bunk, and, with a scream, bring some whiskered relative to her rescue, sends a cold chill of teror down your spine at the very contemplation of such a possibility. But, when she *does* loose her hold after a ten minutes balancing in that ridiculous manner, and plants her robust foot fairly upon your abdomen, using that elastic cushion as a foot-hold for another upward boost,—why, confound it, the position becomes exasperating in the extreme. We will wager a yard of Vienna bread that, the woman we have in our mind's eye, but who, at the critical period mentioned, occupied our real eye, weighed 250 lbs., and that her hose did n't fit her either.

---

DR. ELDRIDGE.—No person in the state—not a a public man—was more widely known than Dr. Eldridge. Sorry, indeed, are we to be compelled to chronicle his death. He was a man of great wealth and possessed a great heart as well, which prompted him to spend largely of his means that the people might enjoy it with him. Always generous to the poor. Regardless of his own losses, even to running his iron works, at a sacrifice of money, that his workmen might not suffer for the necessities of life, which would have occurred had he suspended operations, as did nearly all other operators in iron. Foremost in every undertaking that was calculated to benefit his native city. Ever dispensing a princely hospitality to the strangers who visited it. In short, whatever tended to make our city celebrated abroad, in that was Dr. Eldridge largely concerned. Had he never performed any other act of generosity, the construction of his beautiful park—than which there is none fairer in the land, rendering it a resort for the public, in which everybody was at all times free to go, and there enjoy themselves among the beautiful flowers, pleasant walks and drives, or row upon the bosom of its crystal lake—this magnificent spot is his fitting monument. To die regretted, as did Dr. Eldridge, is to have lived to some purpose. A generous, noble man, one who did much for the comfort and enjoyment of his fellow-men, has departed. The death of such a man is a public calamity.

---

A SORE FINGER.—We have been indulging in the luxury of a sore finger. We say luxury, because the possession of an incapacitated finger is not unattended with advantages. Walking about with a finger in a pint of hot bread and milk, exhibits upon its very surface the evident inability of the hand to which it is attached to perform *manual* labor. No one thinks of asking you to do anything—everybody hastens to help you on with your coat and gloves; while at home, you are a perfect nabob. Wife assists in removing your coat, hangs it up for you, brings gown and slippers and adjusts the easiest chair in the room so as to give you the best light for reading and the most warmth from the glowing grate. In short, you are made as comfortable as possible, without the least exertion on your own part. Per contra, however, we have experienced a few difficulties that detract somewhat from our peace of mind while striving—as must sometimes occur, to help one's self. We have found it extremely perplexing to stir a poultice, in a round pan, upon the stove top, with one hand. The confounded pan will spin round like a top when you attempt the stirring movement, and completely defeat your design. We have also experienced the difficulties attending the pasting of a label,—the opening of a door, with a pitcher of water in your only servicable hand,—the procuring of a cup of water from the pump, while pumping the water and then adroitly catching it with the same hand, before it ceases to run,—the opening of your knife and the sharpening of your pencil, and the putting on of your pantaloons in such a manner as not to have the caudal appendage of your shirt resting in ungainly folds under your short ribs. These are a few of the feats that cannot be accomplished with one hand; try it and see.

---

A PERPLEXED COUNTRYMAN.—Years ago, when a certain editorial friend of ours was a boy, he resided with his parents in a Pennsylvania town, a very pretty place it was, by the way, with the more ancient and imposing buildings, constructed as we now meet with them in that state, located flush with the sidewalks, with a number of doors opening directly upon the walk. In such a house as this, lived our friend,—a large dwelling with parlor, sitting-room, alley door way, and office, all opening in the manner described. To this edifice came a countryman, with oxen and wagon, drawing an immense load of potatoes. Stopping at the first or parlor door, he pulled the bell. Our friend, whom we will call Ben, promptly responded to the call, and to the query of the potato dealer, replied that no potatoes were desired. The farmer urged his oxen a step or two further, and rapped at the next door, and when opened again by Ben, enquired—"Do you want to buy some nice potatoes to-day?" "No, sir," replied our friend, and promptly closed the door. Scarcely had he seated himself, before he was conscious of a rapping at the alley door, leading to the kitchen. At this he also appeared, and, upon opening the door, again received the farmer's query—"Do you want to buy any nice potatoes to-day?" "I believe I informed you twice before, sir, that potatoes were not wanted in this house," replied our somewhat annoyed friend. The farmer looked aloft, to determine if he could, how far that man's house extended, and bravely marched to the next door. This was the office door, where Ben's sire transacted his business, and to which he rushed as speedily as possible that he might again hear the farmer's query and enjoy his discomfiture. Opening the door, there stood the farmer, who commenced his usual—"Do you want to buy—" and then recognizing Ben's face again, completed the sentence with—"See here, Mister, do you own this whole darned street? 'Cause if yer du, I'll jist try the next town." Then, with a "woa, haw," to his oxen, went lazily down the street, scrutinizing the long row of houses, but never stopping to make an inquiry until he had passed well beyond the corner.

---

CHRISTMAS.—It is nearly here again. It seems so short a time since we wrote of the preparations for its celebration, one year ago. How time flies to be sure, and how our gray hairs are increasing by reason of it.

We find, as we approach our residence after e duties of the day have been performed, that a sentinel watches for us at the window,—that mysterious packages and bundles are tossed into some dark closet, while the key shoots the bolt into its place, just as we pass the threshold. All is fluster and confusion, because of the preparations of another Christmas. Some of the children are older than they were a few Christmases ago—consequently, Santa-Claus is invited to present a more plethoric purse than formerly, because of the more costly and useful presents required. Boys and girls at 16, have outgrown toys, and demand more substantial tokens of esteem from the mystic gentlemen. Then, there be younger ones too,—wee ones, that had no existence a few years ago. Their faith in Santa-Claus is firm—and may his mystic majesty long be a hope and comfort to them. Daily, yes, hourly, are they speculating as to what old Santa intends bringing them, and as they even now are speculating upon it, we hear their innocent prattle in the next room, in which their hopes and desires are fully expressed, while right glad are we that Santa-Claus is so near at hand to note every little wish. Bless the little children! How we wish that every mother's son and daughter of them, in all this land, could be made as happy on Christmas morning, as Santa will be sure to make those in the adjoining apartment. It distresses us to think of the poor little waifs who will awake on Christmas morning, to find that Santa-Claus has passed them by; that no token of his appreciation or love has been left to them. How their little hearts must ache at such undeserving neglect! If the Santa-Claus of every family would only remember the children of some poor neighbor, we are sure he would appreciate the joy of his own chosen flock more keenly on Christmas day. Would he not?

Hang up the stockings of the little folks! Let not a bright eye be dimmed by a disappointment. Fill their stockings full, old Santa, and remember that no lovlier sight can be seen, than a happy, laughing band of children, investigating the contents of their stockings, on Christmas morning.

---

THE DOCTOR'S CONSERVATORY.—We found our way into the doctor's new conservatory just in time to overhear a somewhat startling nomenclature for a host of beautiful and rare flowers. The doctor had a guest—one of those inquisitive chaps, who forever was enquiring the names of plants which the doctor himself had not yet learned, being a novice and a beginner in floriculture.

Said the stranger, "doctor what do you call that plant with the large mottled leaves, strongly resembling the shape of a ham!" "O that," says the doctor, stroking his beard and striking an attitude of deep meditation, "that, why, let me see, O, yes, that's the *gluteus maximus*, a new variety, and takes its name from its peculiar shape." "Indeed," said the stranger, "I think I have heard it called a *begonia*." "Likely, but this is a much rarer plant," was the thoughtful answer.

"Now, there's a queer looking flower," again observed the stranger, see how narrow the petals are, and how they terminate in a black clot in the center, why, I declare, it looks very like an eye—what do you call it?" "Yes, that *is* a beautiful flower, and a rare one, too, I'm told. I think Rawson informed me that was a *processi ciliares*, at least, it has that appearance," was the prompt response.

"What a beautiful, beautiful vine that is—with such graceful little curves in it, and the leaves alternating, one on one side, the other just above it on the other, what do you——" "O, that's a *cerebro spinal plexus*," anticipating the question. "Indeed, why it looks something like smilax, does n't it?" "Well, yes, I think it does a little." "What do you call that liver-colored plant with the large leaves with the bluish veins running through it?" "The correct, and I believe generally accepted name for that plant is the *hepaticus*, but the truth is, I don't believe these florists know the meaning of more than half the names they give us. It is my solemn and unbiased opinion that these fellows sit up nights to concoct outlandish names, just to set our teeth on edge when we try to pronounce them. But rest assured, the names I give you are correct—don't think I'll ever forget them, because of their having been so thoroughly impressed upon my mind while in the dissect—I should say, in the labeling room of my father's extensive conservatories, some fifty-five or sixty years ago, that I have never forgotten them, never."

"Now, what in all that's wonderful, causes that vine to run first to the right, then to the left, all the way up to the roof? Trained that way? Ah, ah—what's it's name?" "What the deuce *did* Rawson call that? O, yes, I remember now, that sir, is the *jejunum*, and what is strange, when it gets down this way a few kinks it is then named the *ileum*, and that blossom hanging there that looks like a small gourd, why that's the *appendix vermiformis* of the plant." "Queer to tag on so many names to one poor little plant like that, isn't it?" "I should say so, and how the deuce you can remember them all, is more than I can tell."

"O, look at that, when I touched this plant it drew itself right up in a heap—now that's queer—see there—see it pull itself up—right under your nose, as it were,—now, I would like to know what you call *that?*" "Let me see, it isn't labeled—but I'm quite sure that's the *lavator-labii superioris-alæque-nasi*, unless Rawson deliberately prevaricated, and if he did, he deserves to have his back broken for fooling a chap with such a malicious name as that." "Just write that down for me, please, doctor, I want to get some of the seed, if I can. My wife has a large circle of relatives who make my house a place of unceasing rendezvous. I *would* like to break some of their jaws, if I only could. There, thank you. Where did you say the seed might be had?" "O, send to Vick, he has it, has everything in fact, in the way of seeds—but say, when you get his answer, let me see it, will you?" "O, certainly, good day." "Bye, bye."

